# Migration and Mental Health in Two Contemporary Memoirs

**DOI:** 10.1007/s10912-024-09874-w

**Published:** 2024-07-22

**Authors:** Lena Englund

**Affiliations:** School of Humanities, University of Eastern Finland, Joensuu Campus, Yliopistonkatu 2, 80100 Joensuu, Finland

**Keywords:** Mental health, Mental illness, Memoir, Migration, Refugee, Undocumented, Advocacy

## Abstract

This article examines two autobiographical texts that address the relationship between migration and struggles with mental health: Karla Cornejo Villavicencio’s *The Undocumented Americans* (2021) and Dina Nayeri’s *The Ungrateful Refugee: What Immigrants Never Tell You* (2020). Both memoirs help bring mental health issues to light in situations of precarity, and the texts indicate that it is not just the experience of physical dislocation that may cause or exacerbate struggles with mental health, but the disconnect from other people, from citizenship, and the nation itself. Nayeri and Cornejo Villavicencio do not focus on narratives of recovery or healing but provide space for the experiences of other undocumented migrants trying to navigate the European asylum system or difficulties in obtaining American citizenship. The article argues that the two authors use their experiences of migration and mental illness for greater advocacy purposes with regard to human rights. The struggles with mental health present in the two memoirs intertwine with the treatment of undocumented migrants as described by the two authors, going beyond the personal experience of mental health, or illness, connecting it with migration practices and policies in the United States and Europe.

Current events such as the migrant crisis in Europe in 2015–2016, Australia’s Nauru project, and the Trump administration 2017–2021 (which may be on a repeat trajectory in elections 2024) have made migration a central issue in public debate, bringing to light the mental struggles of people caught up between barriers, in camps, in detention, as undocumented, or trying to come to terms with past experiences of being in flight. The personal narratives emerging engage with current migration policies and practices and shed light on the connection between the precarious migrant experience and mental health. This article engages with two such accounts: Karla Cornejo Villavicencio’s *The Undocumented Americans* (2021), and Dina Nayeri’s *The Ungrateful Refugee: What Immigrants Never Tell You* (2020). Nayeri recounts leaving Iran in 1988, eventually securing asylum in the United States and getting recognized as a refugee. Cornejo Villavicencio’s memoir centers on having arrived undocumented from Ecuador to the United States and later receiving DACA status, which concerns minors whose deportation is deferred but whose citizenship status is not guaranteed. The two memoirs refute the more traditional model of mental illness memoir which often depicts symptoms, diagnosis, treatment, and potentially some form of recovery. Both authors also question the connection between their migration experiences and mental illness diagnoses. The article argues that both authors use their experiences of migration and mental illness for greater advocacy purposes. Issues related to mental health in the memoirs intertwine with the treatment of undocumented migrants as described by the two authors, going beyond personal experience and becoming related to practices and policies in the United States and Europe.

Struggles with mental health become part of the personal narrative and the migration experience in subtle ways, many of which are culturally specific. Donohue-Smith (2011: 142) observes the importance of acknowledging cultural contexts in illness memoirs. Cornejo Villavicencio writes from an American context with an Ecuadorian background, focusing on Latin and South American day laborers, and Nayeri lived for many years in the US after receiving refugee status and ultimately citizenship and then relocated to London. Her Iranian background is significant for her story and for her account of struggles with mental health. The two memoirs can thus be seen as having been produced for an international, English-speaking audience. Both authors became migrants as children, and as research confirms, “pre-migration trauma, forced displacement, and the post-migration environment” affect the mental wellbeing of children and teenagers, leading potentially to a “high prevalence of mental illness” (Blackmore et al. 2020: 712). What sets the two memoirs apart, however, and which justifies choosing them for analysis, is that one represents a so-called success story of getting refugee status and American citizenship, while the other represents experiences of remaining undocumented. The experiences of mental illness are also different, as Nayeri does not explicitly connect her asylum seeker past with the mental health issues she faces, whereas Cornejo Villavicencio makes more straightforward parallels.

Autobiographical writing is a popular genre (see for example Eakin 2020; Rak 2013), reaching large readerships and being in a sense a democratic form of writing. Self-publishing has made it possible for individuals to publicly disseminate their narratives (Laquintano 2016; Hviid et al. 2019). The personal story is also as political as ever, having significant power in terms of those who are marginalized (Gilmore 2019). Experiences of migration and mental illness combine marginalization in two contexts. Previous studies of mental illness in life writing by people who have migrated focus, notably, on gender and race (Grimmer 2020; Seethaler 2021), and the relationship between mental illness, writing, and language (Khakpour 2017). A recent literary project that depicts struggles with mental health among migrants is the *Refugee Tales* volumes, advocating against British practices of indefinite detention for asylum seekers. The volumes have gained significant scholarly attention (see for example Hulme 2020; De Capitani, 2023; Mayer et al. 2023) for their advocacy work in particular.

Autobiographical writing with an advocacy purpose draws on personal stories and “the larger conditions” behind them (Polletta 2023: 109), and in the case of projects such as *Refugee Tales*, the advocacy efforts go beyond individual authors. The memoirs examined engage with “the larger conditions,” yet literary texts may not have much impact on actual policies and laws (Kurz 2015: 19). The individual story may still evoke more empathy than for example statistics (Kurz 2015: 32). Advocacy is relevant from a marketing perspective, too, as such writing can be seen as “both popular and professionalized” (Polletta 2023: 109). Nayer and Cornejo Villavicencio are both professional writers, utilizing their skills to present a narrative that is both personal and political, drawing on a popular genre. Berman (2019: 336) notes that authors of mental illness memoirs become “wounded storytellers engaged in writing/righting wrong.” The memoirs reveal the hardships involved with living as undocumented, and they vocally critique asylum and citizenship policies. Representation becomes a central issue, too, from an ethical perspective as the memoirs are to some extent collective documents, and as observed by Stonebridge (2021: 13), “giving voice” to others in personal accounts may create hierarchies between writers and individuals whose stories are retold. Wilson (2021: 263) adds that “representational appropriation” may occur, particularly if the stories are related by “western writers, editors or translators.” Nayeri and Cornejo Villavicencio engage in advocacy writing not just in terms of mental health but in relation to migration policies and practices, emphasizing the “ungrateful” refugees claiming their place at the table and the undocumented risking deportation. These figures are present in the titles, too, of the two memoirs.

The stigma attached to mental illness to some extent combines with the stigmas attached to being a migrant. The two conditions remain locked in disconnect and uncertainty, relating to personal identity and conceptions of the self as represented in the memoirs. Using terms such as *mental illness* also poses problems, something which Venkatesan and Saji (2021: 145) have observed. Their paper applies the term “to refer to a diagnosed disorder and treats illness as separable from the person who suffers it.” A similar approach is supported by Packer (2017: xx), who argues that the term mental illness “denotes disorders that are identified as illness and are diagnosed as pathological by medical or psychiatric practitioners.” The distinction between health and illness is perhaps nowhere as unclear as in connection to the human mind. Bhugra et al. (2013: 3) argue that *mental health* concerns human relationships and the ability to form them and to regulate actions and feelings. Illness, then, would potentially be the absence of the things listed. Slade (2009: 18) emphasizes knowing the experience of patients, instead of seeing diagnoses as “an authoritative pronouncement.” Lived experience is at the center of autobiographical writing, and Donohue-Smith (2011: 141) observes that memoirs about mental illness portray “a life lived within a web of shifting intrapersonal, interpersonal and environmental forces.” In the texts examined, migration and the outsiderness that comes with it form part of these forces, particularly for those who lack permanent residence.

Mental illness is a secondary storyline in the memoirs, working to highlight the uncertain status of migrants and the struggle involved with relocation and belonging. Green (2021: 629) calls illness and migration in the memoir a “dualist trope of identity,” emphasizing their importance for identity construction. Some caution is necessary, also observed by Donohue-Smith (2011: 138), who notes that memoirs and other forms of personal narration cannot be seen as complete accounts of struggles with mental health, and the two memoirs examined address and portray the self as “fragmented, provisional, multiple, in process” (Baena 2005: 212). Nayeri’s and Cornejo Villavicencio’s memoirs indicate that the fragmentation that can be seen as part of any life narrative is exacerbated by the displacement of both body and mind (Khakpour 2017: 184) and reinforced as advocacy writing through the collective dimension of the two texts.

## Battling the metal bar in *The ungrateful refugee: What immigrants never tell you*

*The Ungrateful Refugee* (2020) consists of several different parts and storylines, recounting Nayeri’s own life and those of asylum seekers in Europe. Nayeri’s earlier novel *Refuge* (2017) is also partly autobiographical (Shymchyshyn and Chernyshova 2022). In the latter half of the memoir, Nayeri outlines travels to camps in Greece in order to reconnect with her own past. Nayeri’s experiences in the asylum system relate to her mother, who converted to Christianity from Islam during a trip to London to visit her mother, Nayeri’s grandmother (Nayeri 2020: 27–28). Nayeri reports having been born in Iran in 1979, growing up in “wartime” (2020: 21). The visit to London in 1985 presents a starting point for Nayeri’s counting, as the family returns to Isfahan after a bullying incident when Nayeri’s finger is partly severed (2020: 31). She is given clothes with which to cover herself: “Nothing was simple or practical; nothing was as I liked. And so, one day in first grade, I started counting things on my lucky fingers” (2020: 34). “Lucky fingers” refers to adult conversations Nayeri overheard since her fingertip could be reattached after the incident (2020: 33).

Struggles with mental health appear in connection with signs of war and the uncertainty amidst which she grew up. Nayeri calls it an “itch in my brain,” emerging at first gently, “whispering that I might take a moment to count my pencils,” and later growing stronger: “Then, that night, it grew bolder, suggesting that the weight of the blanket be distributed evenly along my arms. The itch became a part of me, like the freckle above my lip” (2020: 21). Nayeri does not connect the emergence of these behaviors with the experience of migration, stating explicitly that life as asylum seekers in Abu Dhabi with her mother and brother, or in Italy to which they relocated before being accepted for resettlement by the United States, was not the reason the symptoms appeared but merely made them worse (ibid.). Struggling with mental health in Nayeri’s account becomes exacerbated by migration but is not depicted as induced by it. The passages imply compulsive behavior although this is not directly referred to yet by Nayeri. As defined in psychiatric contexts, “OCD is a psychiatric disorder characterized by distressing intrusive thoughts, urges, or images (*obsessions*) and repetitive behaviors and mental acts aimed at reducing anxiety or distress (*compulsions*)” (Boisseau et al. 2020: 250). They also observe that symptoms of OCD often begin before adulthood, gradually growing more severe (ibid). Symptoms can also begin before puberty (251). Ghose (2023: 27), in her analysis of Nayeri’s memoir, argues that the author “obsessively, compulsively” suggests going back to her past in order to relive the experience of being in a refugee camp, without knowing when waiting will be over, indicating that the writing itself recreates or mimics symptoms connected with OCD.

Nayeri recounts that “the itch endured” even when visiting her father’s relatives, making her “tuck my grandmother’s chestnut hair into her chador with the edges of my hands, circling her face and squeezing her cheeks until I was satisfied” (2020: 21). The “itch” also compels Nayeri to “straighten the papery skin of my ninety-year-old nanny, Morvarid, pressing my palms across her forehead as one would an old letter” (ibid.). Boisseau et al. (2020: 53) confirm that children in particular may have “rituals involving other people (usually family members) and tic-like compulsions,” to which Nayeri’s memoir seems to testify through her behaviors with elderly relatives. A memoir is always retrospective, as the childhood self portrayed by Nayeri is a construction by her adult self, and memoir inevitably represents the time of writing far more than it recreates any past experience. Eakin (2005: 5) argues that the illusion of retrospection relates to transitioning from “the past into the future, and we want to bridge the gap,” and he adds that “the present isn’t a story yet.” Bringing childhood memories into the present gives shape to personal pasts and to the elusive present, made more fragmented in Nayeri’s writing by the stories of people she meets who have “no future” (Nayeri 2020: 172).

The author describes her teachers as “militant women” who were “cruel,” creating a competitive atmosphere with rigid rules (2020: 22). Lists of pupils’ performances in various subjects were routinely posted on the wall, and Nayeri recounts how one of the girls, who often ended up at the end of the list, one day wet herself at her desk (2020: 23). Nayeri refers to the event as the girl’s “quiet surrender,” giving up in the harsh competitive environment in which she was not excelling. Nayeri remembers how she arrived home that day thinking about what had happened in class and counted the books in her shelf: “It wouldn’t be right to count to eleven – I had to count the seven books, then the four. And the next time I bought books, say three of them, I would count the three, the seven and if I still remembered them, the four, each time I left my room” (ibid.). The ritual is not examined beyond the actual act of counting the books, but Nayeri likens the anxiety behind the counting with a “metal bar”: “I breathed deeply until the thing floating too high in my chest (I imagined a metal bar) had moved back down, away from my throat” (ibid.). The counting thus acts as a method to contain the anxiety.

After Nayeri’s parents’ marriage falls apart, partly due to her mother’s activities within her Christian church that get her to get into trouble with authorities, her mother eventually leaves Iran with Nayeri and her brother (2020: 54). They fly to Dubai on tourist visas, with the intent of applying for asylum (2020: 76). The asylum process turns out to be lengthy, and eventually, the Nayeris overstay their visa, becoming “illegal immigrants” (2020: 91). Nayeri becomes acquainted with another family in a similar situation and starts spending time with their daughter Mozhgan who suffers from seizures (2020: 92). Nayeri recounts feeling a connection with the girl, comparing Mozhgan’s seizures to her own need “to feel my chin against my neck or breathe in so deep that the metal bar would move back down into place” (2020: 93). Nayeri states that she “understood the illness” yet felt compelled to leave Mozhgan every time she had an attack. The similarity between the girls, despite their different symptoms, causes Nayeri to distance herself every time an attack occurs. The symptoms of the girls, while different, connect and separate them, indicating that there is a deeper stigma attached to Mozhgan’s seizures. Nayeri (ibid.) observes that she and her brother Daniel were afraid of the girl. Despite these complex feelings, Nayeri also recounts that Mozhgan gave her freedom to be herself: “We were allies in our strangeness” (ibid.).

Despite this bond, Nayeri observes that she knew their symptoms were different because “my thing could be overcome, given time and some breathing room” (2020: 94). Mozhgan’s condition is thus presented as permanent, whereas Nayeri’s behavior is portrayed as manageable, less severe. The memoir implies a certain status difference between conditions, depending on how well they can be managed. No details are revealed about Mozhgan’s seizures and there is no information as to whether they could have been managed with proper treatment and medication, nor is the story told from Mozhgan’s perspective. The subjectivity of Nayeri’s experiences is emphasized. In this context, Donohue-Smith (2011: 140) makes an important observation about personal narratives of mental health issues, arguing that they can be read by lay people as well as those with medical training, yet it is important to bear in mind that memoir does not portray any absolute truths (ibid.). Revisiting the awkward friendship with Mozhgan enables Nayeri to re-evaluate her own symptoms in relation to her childhood, emphasizing the memoir’s temporal trajectory.

The obsessive behavior emerges again when the family moves to another accommodation which has Western toilets, causing Nayeri difficulty as she is not used to them. Her mother scolds her, telling her to “stop this *vasvas*” (2020: 95), to which Nyeri explains the following in parentheses: “The Persian word for OCD is quaint enough for parents to throw around. I hated it, though. My habits were *normal* and I liked them treated as such” (ibid.; emphasis in the original). The mention of OCD indicates a medical diagnosis, at least self-imposed. The comment by her mother implies frustration with the behaviors of which the family was clearly aware, whereas Nayeri underlines that to her they were what she terms normal. Diagnoses are complex, as observed by McNally (2011: 8–9), who states that from a lay perspective, mental illness tends to be equated with schizophrenia, which is not the most common disorder. McNally suggests that “mental illness occurs on a spectrum of severity” (9) and notes that the criteria behind medical diagnoses lack “social context” (12). However, as observed by Green (2021: 639), diagnoses also help provide “shape and narrative to an experience of illness,” and Stanger Elran (2019: 1) adds that the narratives of psychosis she has examined offer novel interpretations of “self and personhood.” Memoir of migration and mental illness gives shape to both experiences, and diagnosis can here be a device for writers with which to frame their personal narratives.

Nayeri’s family ends up in Italy at a camp for asylum seekers, located in an old hotel in the countryside (2020: 106), eventually receiving asylum and refugee status in the United States in 1989 (2020: 113–114). After some struggles in the beginning, Nayeri sets her mind upon Harvard, thinking that she will find her community and sense of belonging there, among other “strange girls who count everything, who maybe have a raw spot on their neck” (2020: 285). The line indicates a continuous search for community, for belonging that goes beyond the migration experience, and the struggle with obsessive compulsive behavior is presented as a potentially shared experience. The same search is present in Nayeri’s visits to camps for asylum seekers, asking herself whether it is “cruel for a person who’s come unstuck to return to another’s purgatory” (2020: 181). Eventually, Nayeri gets accepted to Princeton, and the personal story of growing up effectively ends there (2020: 299). Later in the memoir, Nayeri remembers the interviews the family took part in during the asylum process and argues that children “instinctively know how to tell good stories, especially true ones” (2020: 238), indicating that the asylum interview was an easier procedure for her than for adults. She writes that the interview did not result in compulsive and obsessive behavior, yet when compared to another interview, relating to a writers’ workshop in Iowa, she had to “hold in the tics” (2020: 239). Before arriving in Iowa, Nayeri recounts that her “physical symptoms of the OCD returned. […] As in Iran, a necessary part of me was stifled” (2020: 229–230). The memoir connects the adult self with childhood, suggesting that symptoms of OCD emerge in the vacuum that follows the experience of not being entirely settled, and not having a clear direction into the future.

Just as Mozhgan provides a mirror for the child self, a similar comparison emerges in adulthood. Nayeri recounts reconnecting with her grandmother some months before she traveled to Greece in 2017 to visit camps for asylum seekers. Nayeri addresses her grandmother’s life story, who got married as a teenager and left her husband for someone else with whom she was supposed to start a new life in London (2020: 127). The man never showed up, leaving her grandmother waiting for decades (2020: 129). Later, she moved constantly, compelled by what she felt were unfriendly neighbors who kept causing disturbances: “In each new flat, her hope returned for a time, the itch in her brain calmed” (2020: 131). The constant relocation is thus presented by Nayeri as an attempt to calm the “itch,” as a way of coping with anxiety about a life that never materialized. Nayeri observes that she understands her grandmother better than any other family member (2020: 132), and this understanding forms a connection: “She is me in old age. I can see her struggling with the itch in her brain. […] Are there homes that I have rejected because the itch wants me to be ever on the move?” (2020: 132–133). The passage implies that the “itch” causes restlessness and prevents belonging, creating a life of constant relocation and movement. It is not presented as much as connected with the migration experience per se, or the refugee condition, but with a more encompassing lack of roots and belonging. Nayeri returns to the need for constant movement later in the memoir, writing that she, too, has moved frequently: “This isn’t displacement anymore. It’s compulsion” (2020: 183). The comment implies that the need to keep moving is more connected with the itch than the other way around, suggesting that the experience of displacement is secondary to the need to relocate.

Research on mental illness shows that particularly PTSD, post-traumatic stress disorder, is more prevalent among those categorized humanitarian migrants (Cooper et al. 2019: 2). Nayeri’s memoir discloses several encounters with migrants and people working with asylum seekers, and some of these encounters reveal the precarious mental stability of people living in camps. The decision to visit camps is defined by Nayeri as related to how “the world is turning its back on refugees” and to sort out her own identity as she has now become a mother to her daughter Elena (2020: 139). Nayeri questions her decision to return: “What if I’m stuck at the airport? What if my passport gets stolen? What if I’m detained? Where is my Elena?” (ibid.). The questions indicate intrusive thought patterns, and the personal experience of displacement is represented as never-ending, despite American citizenship and a successful professional life, and despite having become “unstuck.”

One of the main parallel stories told by Nayeri concerns that of Kambiz, who left Iran as a young adult (2020: 55), arriving in the Netherlands and seeking asylum. His application was rejected, yet he continued to work illegally (2020: 219), eventually spending a year in detention after which he “became depressed and began to dream of death” (2020: 221). Nayeri calls the endless waiting involved with asylum seeking “[m]adness in increments, by an ever-shifting endpoint” (2020: 223), like a mirage that keeps moving further and further away. In the case of Kambiz, the endpoint is tragic, as he eventually killed himself, setting himself on fire in Amsterdam (2020: 225). The culmination of Kambiz’s distress is irreversible, and Nayeri ponders the meaning and futility of his death, wondering why he chose such a way to die: “Maybe he wanted to remain in the country’s psyche, […] to burn his image into their memories” (2020: 227). The desperate act thus comes to symbolize not just the “madness” of Kambiz himself and the decision he made, but also the “madness” and decisions made by the country to which he had relocated. This comes to represent the asylum process and the indignity of waiting, as they intertwine with personal suffering and tragedy in Nayeri’s memoir. Another incident concerns a mother asking Nayeri if she could take the woman’s son with her to England to pursue a better life there, to which Nayeri reacts by asking whether the woman is “crazy” (2020: 180). The madness of waiting transforms into desperate acts as represented in the memoir. The inclusion of other stories than her own reinforces her critique of the asylum system, and Cornejo Villavicencio does similar work in her memoir, including the voices of so many other undocumented migrants and the ways in which they are treated.

## Traumas of separation in *The undocumented Americans*

*The Undocumented Americans* (2021) offers a portrayal of what it means to live without citizenship status in the United States from perspectives that are both deeply personal and advocacy-oriented. Villavicencio, writer and journalist, builds a multifaceted narrative of undocumented status through interviews with migrants across the US, stating that she hopes to “write from a place of shared trauma, shared memories, shared pain” (2021: xvi), indicating a collective purpose. She also asserts that in order to write her book “you have to be a little crazy” (2021: xv), and she (ibid.) sees herself as “crazy enough” for the job. “Crazy” here may refer to courage, or certain foolhardiness, in taking on such a politically contested topic. Cornejo Villavicencio states that the way migrants are often portrayed is through tales of so-called dreamers, undocumented people who arrived in the United States as minors. She hopes to provide stories of people who often work as laborers or housekeepers, “the people underground” (2021: xvi-xvii), in order to make sure that they are “not effaced from public record” (Carpio 2023: 226). As observed by Boelhower (2005: 222), autobiographical writing by people who have immigrated to the United States often critically scrutinize society and politics, due to their own precarious position. Mental health issues remain a central topic throughout the memoir, both in terms of the author’s personal life as well as the people she encounters. The word “trauma” is invoked in the introduction and referred to several times in the text, indicating that the unsettled status of undocumented migrants has profound consequences for their mental wellbeing.

Goodman et al. (2017: 309) study trauma among refugee women and women with undocumented status, stating that from a medical perspective, it is often seen through the lens of PTSD, which is defined as involving “intrusion symptoms, avoidance, negative mood and cognitions, and arousal symptoms” (315). They also report that many women experienced nightmares. A broad perspective on trauma is necessary for Cornejo Villavicencio’s memoir, which recounts the stories of people in complex situations, living lives of multiple struggles. The status of being undocumented is important for mental health, and not just in terms of what the person has experienced. Gonzales et al. (2013: 1183) report in their study of young people with undocumented status that participants had trouble “achieving a sense of coherence and continuity,” which are important for “a healthy identity.” Certain disconnect emerges in the memoir in relation to Cornejo Villavicencio and her own family, particularly in terms of her early childhood.

Cornejo Villavicencio reveals that her parents, of Ecuadorian origin, originally decided to move to the United States for financial reasons. They first left Villavicencio behind, who was just a year and a half old (2021: 4), and brought her to join them when she was four, in order to give her the prospect of a better education (2021: 5). Cornejo Villavicencio discloses that her parents continued to struggle with money and with paying her tuition fees but that she had benefactors whom she compares to Gertrude Stein and herself to Hemingway, writing that “I was *Van Gogh*, crazy and broken” (2021: 6). The reference to “crazy” yet again implies something slightly different from the previous passages in which the word was used. Van Gogh likely refers to the painter who allegedly severed his own ear as an act of self-harm and whose psychiatric challenges have been well researched (Smith et al. 2023: 1). Ernest Hemingway, too, suffered from struggles with mental health (Hutchisson 2016: 2), eventually killing himself (248). Comparisons to these iconic artists indicate that Cornejo Villavicencio draws on her struggles in her writing, even seeing them as a source of strength. That is not to underplay the debilitating effects of mental health issues, some of which are outlined in painful detail by Cornejo Villavicencio, nor is it meant to emphasize any views of “artists as tortured souls” (Kaufman 2014: 400), a topic of some debate. McNally (2011: 30) asks if adequate medication would have “blunted the genius of van Gogh, Beethoven, and Virginia Woolf,” whereas Berman (2019: 18) asserts that illness relates to creativity. In the case of the two memoirs examined, they are creative texts in which mental illness is a significant theme. Another study of the relationship between creativity and mental illness confirms the hypothesis that some “vulnerability to or low levels of psychopathology” connect with creativity, whereas the connection weakens with more severe mental illness (Parnas et al. 2019: 362).

The reference to Cornejo Villavicencio being “crazy and broken” indicates mental struggle already in early life. Much of her memoir revolves around her relationship with her father, whose struggles she outlines in detail and elaborates on their relationship when she was growing up. Cornejo Villavicencio reveals that her father lost his driver’s licence in the aftermath of 9/11 when policies became stricter for undocumented people. Her father cried after having his licence suspended, an emotional scene to which the author was unused. Cornejo Villavicencio defines both herself and her father as “difficult,” but states that she was also “dark and precocious” (2021: 41). This precociousness became visible, according to Cornejo Villavicencio, in a “my immigrant-third-grader-is-reading-Hemingway-but-is-secretly-drinking-Listerine-and-toothpaste-until-she-throws-up-because-she-wants-it-to-kill-her kind of way” (2021: 41–42). Cornejo Villavicencio therefore seems to refute views of the suffering artist, the “tortured soul,” revealing the debilitating and dangerous consequences of her symptoms as the passage indicates self-harm and potentially even suicidal ideation at a young age. To help his daughter, Cornejo Villavicencio recounts that her father created a busy schedule for her to keep her away from herself. Memoir can to some extent be seen as performing similar tasks, creating distance to self through autobiographical narration, and the collective dimension gives personal experiences a wider context.

Another connection between her father and her struggles with mental health appears when Cornejo Villavicencio notes that her father had beautiful handwriting (2021: 51–52), as did she, too, “for a long time until I started taking lithium and then my hand shook bad. My dad was so sad” (2021: 52). The *NHS* reports that lithium is used to treat conditions such as bipolar disorder, depression, and mania (“Lithium”) (Lithium 2020). The connections between Cornejo Villavicencio’s mental health issues and her father also come to encompass the United States themselves. She argues that the American government has committed “crimes against immigrants” (2021: 60) and that these things were happening already when she was young, writing that she “felt crazy for thinking we were under attack, watching my neighbors disappear and then going to school and watching the nightly news and watching award shows and seeing no mention” (ibid.). Cornejo Villavicencio here seems to use the word crazy to describe her lived reality and those of other migrants, invisible to so many.

Eventually, Cornejo Villavicencio states: “I am also crazy. Pero why? My diagnoses are borderline personality disorder, major depression, anxiety, and OCD. (I love diagnoses. Gives you the ability to read about yourself.)” (2021: 61). “Crazy” in this context becomes connected with Villavicencio’s diagnoses. As argued earlier, diagnoses can give “shape and narrative to an experience of illness” (Green 2021: 639). In Cornejo Villavicencio’s account, diagnoses provide shape and narrative to her conception of self. Brewis and Wutich (2020: 152–153) discuss ways in which to diminish the stigma surrounding mental illness, and they argue that publicly known figures can help do this, inspiring “public debate and reflection.” Although Cornejo Villavicencio may not be as well known as some of the celebrities listed by Brewis and Wutich, her platform is significant. The work she performs through her writing is not centered on mental illness, but on undocumented migration and the lives of those who live in the margins, yet with the explicit aim not to victimize them (Carpio 2023: 223). Incorporating experiences of mental illness works to highlight both the internal and external struggles of migrants in precarious situations. Further, Brewis and Wutich observe that some celebrities open up about the “complicated, chaotic, ongoing challenges of living with serious mental illness” (154). Cornejo Villavicencio, too, can be said to perform such work through her memoir which shows the less heroic side of mental illness.

When listing her diagnoses, Cornejo Villavicencio argues that research indicates that “the flooding of stress hormones resulting from a traumatic separation from your parents at a young age kills off so many dendrites and neurons in the brain that it results in permanent psychological and physical changes” (2021: 61). Muñiz de la Peña et al. (2019) report on the effects on children who were separated from their parents as a result of the zero-tolerance policy enforced in 2018 during the Trump administration (154). Children were separated from parents sometimes for as long as months (155). While Muñiz de la Peña et al. (2019: 160) note that it is hard to determine what the long-term results of the policy will be, their findings indicate that “severe traumatic impact occurs with likely enduring consequences.” A similar conclusion is provided by Rollins (2018: 161–162), who speaks of “psychological damage” resulting from the separation. She mentions “toxic stress” (161), reinforcing Cornejo Villavicencio’s statement, who refers to herself and other children who have been and continue to be separated from their parents as an “army of mutants” with brains that have been permanently altered (2021: 61). She asks “what will happen to us” (ibid.), implying a sense of isolation and separation.

The “us,” the “army of mutants,” and particularly the question about who will take care of migrants emerge in different contexts in Cornejo Villavicencio’s memoir. They come to encompass more than just children separated from parents, covering also undocumented migrants who experience separation in multiple ways, sometimes from themselves. Many of the day laborers Villavicencio meets worked with the World Trade Center cleanup, struggling with various health issues afterward. One of them is Enrique, who gets flashbacks from the work he did, once about being “surrounded by dank water, in the dark, next to fallen lights, shattered glass, and ash, the strong smell of mildew and chemicals soaking fabrics and furniture” (2021: 35). The flashback caused him to fall off a ladder and break his arm. He attends a support group for people suffering from various conditions such as “PTSD, anxiety, depression, and paranoia” (2021: 34). Cornejo Villavicencio discloses that she knows about the mental health problems of the people who attend the group, and she asks them what medication they take and what symptoms they have but get somewhat reluctant responses: “In our community, there is an ingrained idea that if you are sick, it’s a weakness, a symptom of our internalized bootstraps mythology. ‘You don’t have to pretend with me,’ I say. We end up confiding in each other” (2021: 36). The comment indicates a certain cultural stigma related not just to mental illness but any illness at all and also shows that Cornejo Villavicencio is able to overcome it in the group through sharing her own experiences of struggling with mental health issues. Her memoir can be seen as working to similar ends. Seethaler (2021: 71) notes that for many people living in the margins, depression may not be seen as a “legitimate disease.” While the conditions listed in the memoir examined are varied, a similar sentiment can still be detected, further emphasizing the importance of nonfictional texts such as *The Undocumented Americans*.

Another cleanup worker is called Paloma, whose story works to offer a different perspective on the topic of separation. Paloma is recounted as having left Colombia to get away from her unfulfilling life there, admitting for example that she did not particularly enjoy being a mother or a grandmother, or babysitting her grandchildren (2021: 45–46). She also wanted to escape the fate of people close to her: “Paloma tells me her brother drowned himself, one of her cousins hanged himself, and another threw himself off a tall building in Bogotá. She wonders: If she had stayed in Colombia, would she have followed suit?” (2021: 46). Paradoxically, the act of leaving and migrating can thus be seen as an attempt to *preserve *mental health, although Cornejo Villavicencio reveals that Paloma suffers from “sleep apnea, PTSD, depression, anxiety, gastrointestinal issues” (2021: 45) along with breast cancer (ibid.). The story of Paloma serves as a reminder that there is no common narrative of migration, that backgrounds and experiences, future hopes and dreams, are personal and individual, and that these deserve recognition also when addressing wider structures and policies.

The Victim Compensation Fund, which provides compensation for victims of 9/11, exemplifies such a structure in the memoir and is reported by Cornejo Villavicencio as having demanded proof of work at the cleanup site. She is critical of this approach, stating that undocumented workers would not be in the possession of such papers as their presence and work at the site would not have been documented anywhere (2021: 49). The paperless state of undocumented migrants thus extends beyond residence and citizenship. The difficulty in getting access to compensation is repeated in the chapter titled “Miami” in which Cornejo Villavicencio visits pharmacies attended by undocumented migrants who do not have access to health insurance. Cornejo Villavicencio also visits so-called botanicas that sell alternative remedies and she criticizes those who see them as “charmingly idiosyncratic” (2021: 67). She asserts that there is a difference between having the choice to use herbal remedies or Western medicine, or not having any access to treatment. These concerns are confirmed by Castañeda (2019: 148), who states that legal status impacts access to health insurance and observes that lack of access to proper healthcare and treatment has led to increased trade in prescription medicine that may be provided “unlawfully and in an unregulated manner” (155). A pharmacy is willing to sell the author medicines such as Risperdal or Seroquel, “powerful antipsychotics. They’re tough drugs. Mean” (2021: 71). Cornejo Villavicencio lists potential side effects and expresses concern about such drugs being sold without any follow-up.

*The Undocumented Americans*, while bringing attention to the struggles and suffering of people without papers, makes a significant, albeit more subtle, effort to increase awareness and acceptance of mental illness. At the end of the memoir, Cornejo Villavicencio reveals that in addition to other conditions previously listed, she also suffers from an undefined ulcer which “hurts the most when, after a long day of reading about people forming human chains to block ICE officers from arresting a man and his child, I sit down to write about my parents” (2021: 155). The passage indicates a connection between the physical and psychological processes, and the author relates her physical illness to symptoms of other migrants: “We’re all fucking *sick*” (ibid.; italics in the original). Just like her use of the word crazy implies many different meanings, “sick” in this context suggests that more is at play than mere physical or psychological symptoms. Throughout her memoir, Cornejo Villavicencio’s personal story alternates with that of her father, of the people she meets, and eventually with the “craziness”, or “sickness”, that encompasses them all and binds them together, struggling for recognition and documentation in a nation represented as reluctant to provide either.

## Conclusion: Advocating migrants’ rights to healthcare and citizenship

Mental health connects with migration in multiple ways in the two memoirs examined but is also examined in separation from it by the two authors. The memoirs open up for further examination of the connection between migration and mental health, particularly in precarious situations where citizenship is unobtainable. Neither Dina Nayeri nor Karla Cornejo Villavicencio offer narratives of recovery or redemption. While Nayeri remains focused on the European asylum system and the suffering of people within it, Cornejo Villavicencio critiques American treatment of people with undocumented status and lack of access to proper healthcare. The “itch” in Nayeri’s memoir is connected to displacement and constant movement, preceding it yet also becoming exacerbated by it, indicating a complex relationship between migration and mental health that is further exemplified by the tragic story of Kambiz who ended his own life. Cornejo Villavicencio uses her multiple diagnoses to highlight the role they can play for personal narration and understanding of the self, working to remove stigma, also addressing the relationship between childhood abandonment and mental illness. The abandonment is presented as carrying on into adulthood, exacerbated by authorities enforcing migration policies.

Instead of becoming solely a separating experience, of othering and outsiderness, mental illness is, paradoxically, presented as a force that can bring people together, without heroic undertones. It becomes a thread told alongside experiences of migration, sometimes overlapping and sometimes in tandem, yet never losing sight of the challenges involved. The separation between health and illness is also questioned in both memoirs, as both Nayeri and Cornejo Villavicencio focus on the human connections they make and revisit in their memoirs, such as the childhood friend Mozhgan and Nayeri’s grandmother, and Cornejo Villavicencio’s father who tried to keep the author from harming herself in youth. Cornejo Villavicencio manages to overcome cultural barriers when talking to day laborers and listening to their experiences of being mentally unwell, also sharing her own. The “army of mutants” in her memoir indicates rebellion against a system that does not look after its most vulnerable members. Cornejo Villavicencio’s questions about who will take care of her and others in a similar situation come to encompass more than just children separated from parents, covering also undocumented migrants who experience separation in multiple ways, even from themselves. Nayeri and Cornejo Villavicencio engage in advocacy writing in relation to migration policies, evoking figures of the asylum seeker in endless limbo and the undocumented worker risking deportation, in order to emphasize collective responsibility. There is no straightforward road to recovery in the memoirs, and, in the personal narratives recounted, this comes to include the asylum system in Europe and citizenship policies in the United States.
